# Emergency Department Visits for Suspected Suicide Attempts — United States, 2021–2025

**DOI:** 10.15585/mmwr.mm7524a1

**Published:** 2026-06-25

**Authors:** Bhavna Singichetti, Alison L. Cammack, Patrick Rekieta, Amy Schumacher, Katharina L. van Santen, Wojciech Kaczkowski, Lakshmi Radhakrishnan, Kayla N. Anderson, Michael F. Ballesteros, Robin Lee

**Affiliations:** ^1^Division of Injury Prevention, National Center for Injury Prevention and Control, CDC; ^2^Office of Strategy and Innovation, National Center for Injury Prevention and Control, CDC; ^3^Epidemic Intelligence Service, CDC; ^4^Detect and Monitor Division, Office of Public Health Data, Surveillance, and Technology, CDC; ^5^ICF International, Inc., Reston, Virginia.

**Summary**

**What is already known about this topic?**

In 2024, approximately 49,000 persons died by suicide in the United States, and an estimated 2.9 million persons aged ≥12 years reported attempting suicide.

**What is added by this report?**

During 2021–2025, numbers of emergency department (ED) visits for suspected suicide attempts and the proportions of those visits among all ED visits for any reason (visit proportions) were highest among adolescents aged 12–17 years compared with other age groups. Overall visit proportions decreased, with the largest decreases occurring among adolescents aged 12–17 years and among females; however, visit proportions increased among adults aged ≥26 years.

**What are the implications for public health practice?**

Timely monitoring and a comprehensive approach that both prevents suicidal behavior and supports persons who have attempted suicide are critical for all groups, especially those with high or increasing numbers and proportions of suicide attempts.

## Abstract

Suicide is a substantial public health problem. In 2024, approximately 49,000 persons died by suicide in the United States, and an estimated 2.9 million persons aged ≥12 years reported having attempted suicide. Emergency department (ED) visits for suspected suicide attempts among adolescents increased during 2020–2021, then decreased in 2022; reports based on more recent data, including ED visits for suicide attempts among older age groups, are lacking. National Syndromic Surveillance Program data were examined overall and by sex and age group to identify changes in ED visits for suspected suicide attempts. During 2021–2025, numbers of ED visits for suspected suicide attempts and proportions of those visits among all ED visits for any reason (visit proportions) were highest among adolescents aged 12–17 years compared with other age groups and were higher among females than among males. Compared with 2021, overall visit proportions in 2025 declined 7.0%, with the largest decreases occurring among adolescents aged 12–17 years (20.8% decline) and females (10.7% decline). Visit proportions increased among adults in age groups ≥26 years (range = 1.4%–15.2%). These findings highlight the need for suicide prevention in all groups, particularly in those with high or increasing proportions of suicide attempts. Timely monitoring of suicide-related data and a comprehensive approach that both prevents suicidal behavior by addressing multiple risk and protective factors and also supports those who have attempted suicide are critical for saving lives.

## Introduction

During 2024, a total of 48,824 persons in the United States died by suicide (*1*). These deaths are only one part of a larger public health problem; an estimated 2.9 million persons in the United States aged ≥12 years reported attempting suicide in 2024^†^ (*2*). Emergency departments (EDs) are an important setting for tracking trends in suicidal behavior in near real-time.

During 2019–2022, notable changes in ED visits for suspected suicide attempts among adolescents and young adults occurred (*3*,*4*). Although ED visit numbers for suspected suicide attempts initially decreased during spring 2020 compared with spring 2019, the number of such visits increased in adolescents by summer 2020; proportions of all ED visits for suspected suicide attempts among adolescents and young adults were elevated through spring 2021 (*3*). A second study of suicide attempts among adolescents found decreases from 2021 to 2022, particularly among girls (*4*). Reports based on recent data on suicide attempts, including those among adults aged ≥26 years, are lacking. To understand whether decreasing trends have persisted and to guide suicide prevention activities, this report evaluated January 2021–December 2025 data from the National Syndromic Surveillance Program (NSSP) on ED visits for suspected suicide attempts, by sex and age group.

## Methods

### Data Source

NSSP receives deidentified ED data from state and local health departments, usually within 24 hours of a patient’s visit to a medical facility. Approximately 83% of U.S. EDs contribute to NSSP. Data filters were applied to ensure that only facilities with rigorous and consistent reporting to NSSP across the study period were included. Among 5,398 facilities that shared data with CDC during 2021–2025, a total of 3,184 (59.0%) met the inclusion criteria after applying data quality filters. These facilities account for 80.6% of ED visits sent to NSSP during the analysis period.

### Data Analysis

For this analysis, a validated definition that excludes visits for nonsuicidal self-harm was used to identify ED visits for suspected suicide attempts (Suicide Attempt Definition Factsheet and Technical Brief | CDC). NSSP was queried for ED visits for suspected suicide attempts and total ED visits during January 1, 2021–December 31, 2025. To account for fluctuations in ED visit volume during the study period, proportions of suspected suicide attempts per 10,000 ED visits for any reason (visit proportions) were calculated overall, by sex, and by age group (≤11, 12–17, 18–25, 26–34, 35–44, 45–54, 55–64, and ≥65 years). Visits with no reported patient age (0.6% of visits) and those with no reported patient sex (0.2% of visits) were excluded from respective age or sex stratifications. Consistent with previously published methods (*3*,*4*), comparisons were made using visit ratios (VRs)^§^ with 95% CIs and percent changes in visit proportions.^¶^ All analyses were completed in R (version 4.4.0; R Foundation). This activity was reviewed by CDC, deemed not research, and conducted consistent with applicable federal law and CDC policy.**

## Results

### Overall Number of Visits and Visit Proportions

During January 1, 2021–December 31, 2025, among 527,183,306 ED visits for any reason, 833,335 ED visits for suspected suicide attempts were identified (15.8 per 10,000 visits); 502,452 (60.3%) of such visits were by females (17.4 per 10,000 visits); and 328,962 (39.5%) were by males (13.8 per 10,000 visits). By age group, the highest number of visits for suspected suicide attempts were by adolescents aged 12–17 years (207,028 visits; 24.8%); this group also had the highest visit proportion of ED visits for suspected attempted suicide (82.2 per 10,000 visits), particularly among girls (120.0 per 10,000 visits). Adolescents aged 12–17 years also consistently had the highest proportions of such visits each month, although visit numbers and especially visit proportions declined during the study period (Figure).

**FIGURE:**
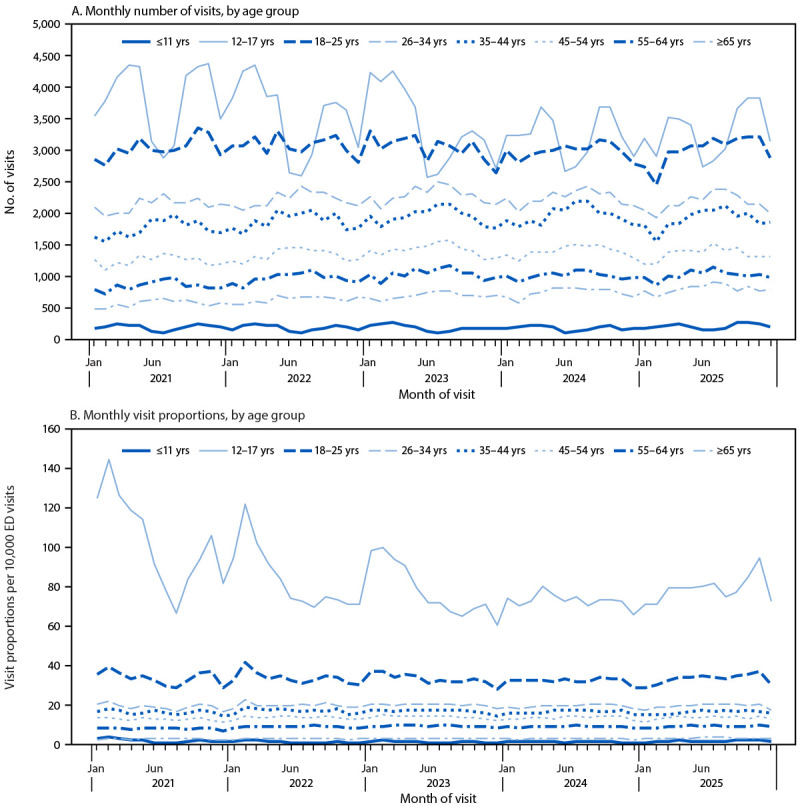
Number of monthly emergency department* visits^†^ for suspected suicide attempts (A) and visit proportions for suspected suicide attempts (B) per 10,000 emergency department visits for any reason, by age group and month — National Syndromic Surveillance Program, United States, 2021–2025 **Abbreviations:** ED = emergency department; NSSP = National Syndromic Surveillance Program. * Filters were applied to only include data from facilities with a coefficient of variation ≤40 and an average weekly informative discharge diagnosis ≥70% for the entire study period. Of 5,398 facilities that shared data with CDC during 2021–2025, 3,184 facilities (59.0%) met the inclusion criteria after applying data quality filters. These facilities account for 80.6% of ED visits sent to NSSP during the analysis period. † CDC Suicide Attempt (v2) | Definition Factsheet & Technical Brief

### Annual Comparisons

Overall, whereas monthly average numbers of ED visits for suspected suicide attempts were similar in 2021 (monthly average = 13,668) and 2025 (monthly average = 13,889), visit proportions for suspected suicide attempts decreased by 7.0%, from 16.6 to 15.5 per 10,000 visits (VR = 0.93) (Table). Compared with 2021, the largest decreases in 2025 occurred in females (VR = 0.89; percent change = 10.7%) and adolescents aged 12–17 years (VR = 0.79; percent change = 20.8%). Among adolescents aged 12–17 years, decreases were larger for females (VR = 0.78; percent change = 22.3%) than for males (VR = 0.91; percent change = 8.9%) (Supplementary Table). In addition, the decrease from 2021 to 2025 in this age group was smaller than the decrease from 2021 to 2024 (VR = 0.74; percent change = 26.5%), indicating a small increase from 2024 to 2025; increases from 2024 to 2025 were also observed for children aged ≤11 years (Table). Visit proportions for suspected suicide attempts by adults aged ≥26 years increased significantly for most age groups during 2022–2025 compared with 2021. In 2025 compared with 2021, increases were observed among adults aged 55–64 years (VR = 1.15; percent change = 15.2%) and ≥65 years (VR = 1.12; percent change = 12.3%). Similar patterns of decreases among adolescents aged 12–17 years and females, and increases in numbers of ED visits for suspected suicide attempts among adults aged ≥26 years were observed.

**TABLE:** Number of visits, visit proportions, visit ratios, and change in visit proportions in emergency department visits* for suspected suicide attempts,^†^ by year, age group, and sex — National Syndromic Surveillance Program, United States, 2021–2025

Characteristic	Average monthly no. of ED visits for suspected suicide attempts^§^	Average monthly no. of ED visits for any reason^§^	Annual visit proportions for suspected suicide attempts, per 10,000 ED visits for any reason	Visit ratio (95% CI)^¶^	Percent change in annual visit proportions compared with 2021**
**Year**					
2021	13,668	8,210,951	16.6	Ref	Ref
2022	13,927	8,728,823	16.0	0.96 (0.95−0.97)^††^	−4.1
2023	14,104	8,949,090	15.8	0.95 (0.94−0.95)^††^	−5.3
2024	13,857	9,068,822	15.3	0.92 (0.91−0.92)^††^	−8.2
2025	13,889	8,974,256	15.5	0.93 (0.92−0.94)^††^	−7.0
**Sex**					
Female					
2021	8,404	4,461,529	18.8	Ref	Ref
2022	8,452	4,756,740	17.8	0.94 (0.94−0.95)^††^	−5.7
2023	8,519	4,896,000	17.4	0.92 (0.92−0.93)^††^	−7.6
2024	8,215	4,967,052	16.5	0.88 (0.87−0.89)^††^	−12.2
2025	8,281	4,924,062	16.8	0.89 (0.89−0.90)^††^	−10.7
Male					
2021	5,235	3,725,127	14.1	Ref	Ref
2022	5,447	3,954,674	13.8	0.98 (0.97−0.99)^††^	−2.0
2023	5,551	4,037,862	13.7	0.98 (0.97−0.99)^††^	−2.2
2024	5,614	4,086,977	13.7	0.98 (0.97−0.99)^††^	−2.2
2025	5,567	4,032,254	13.8	0.98 (0.97−0.99)^††^	−1.7
**Age group, yrs**					
≤11					
2021	195	905,313	2.2	Ref	Ref
2022	184	1,152,013	1.6	0.74 (0.70−0.79)^††^	−25.8
2023	187	1,124,019	1.7	0.77 (0.73−0.82)^††^	−23.0
2024	182	1,089,062	1.7	0.78 (0.73−0.82)^††^	−22.4
2025	209	1,024,123	2.0	0.95 (0.90−1.00)	−5.3
12–17					
2021	3,800	381,394	99.6	Ref	Ref
2022	3,540	427,278	82.9	0.83 (0.82−0.84)^††^	−16.8
2023	3,391	433,541	78.2	0.79 (0.77−0.80)^††^	−21.5
2024	3,225	440,170	73.3	0.74 (0.73−0.75)^††^	−26.5
2025	3,295	417,568	78.9	0.79 (0.78−0.80)^††^	−20.8
18−25					
2021	3,030	901,094	33.6	Ref	Ref
2022	3,078	908,498	33.9	1.01 (0.99−1.02)	0.8
2023	3,044	909,235	33.5	1.00 (0.98−1.01)	−0.4
2024	2,990	914,891	32.7	0.97 (0.96−0.99)^††^	−2.8
2025	3,003	891,690	33.7	1.00 (0.99−1.02)	0.2
26−34					
2021	2,132	1,105,516	19.3	Ref	Ref
2022	2,219	1,106,705	20.0	1.04 (1.02−1.06)^††^	4.0
2023	2,288	1,124,130	20.4	1.06 (1.04−1.07)^††^	5.6
2024	2,244	1,133,131	19.8	1.03 (1.01−1.04)^††^	2.7
2025	2,167	1,094,934	19.8	1.03 (1.01−1.04)^††^	2.6
35−44					
2021	1,753	1,066,530	16.4	Ref	Ref
2022	1,882	1,084,368	17.4	1.06 (1.04−1.08)^††^	5.6
2023	1,954	1,131,362	17.3	1.05 (1.03−1.07)^††^	5.1
2024	1,965	1,160,538	16.9	1.03 (1.01−1.05)^††^	3.0
2025	1,909	1,145,749	16.7	1.01 (1.00−1.03)	1.4
45−54					
2021	1,249	957,694	13.0	Ref	Ref
2022	1,338	960,735	13.9	1.07 (1.04−1.09)^††^	6.8
2023	1,421	988,055	14.4	1.10 (1.08−1.13)^††^	10.3
2024	1,402	1,000,079	14.0	1.07 (1.05−1.10)^††^	7.5
2025	1,360	985,064	13.8	1.06 (1.04−1.08)^††^	5.9
55−64					
2021	852	1,024,512	8.3	Ref	Ref
2022	973	1,046,374	9.3	1.12 (1.09−1.15)^††^	11.8
2023	1,038	1,068,765	9.7	1.17 (1.14−1.20)^††^	16.8
2024	1,013	1,077,546	9.4	1.13 (1.10−1.16)^††^	13.1
2025	1,021	1,066,350	9.6	1.15 (1.12−1.18)^††^	15.2
≥65					
2021	572	1,819,254	3.1	Ref	Ref
2022	636	1,996,054	3.2	1.01 (0.98−1.05)	1.5
2023	693	2,111,587	3.3	1.04 (1.01−1.08)^††^	4.4
2024	744	2,208,088	3.4	1.07 (1.04−1.11)^††^	7.1
2025	803	2,275,042	3.5	1.12 (1.09−1.16)^††^	12.3

**Abbreviations:** ED = emergency department; Ref = referent group.

* Filters were applied to only include data from facilities with a coefficient of variation ≤40 and an average weekly informative discharge diagnosis ≥70% for the entire study period. Of 5,398 facilities that shared data with CDC during 2021–2025, a total of 3,184 facilities (59.0%) met the inclusion criteria after applying data quality filters. These facilities account for 80.6% of ED visits sent to the National Syndromic Surveillance Program during the analysis period.

† CDC Suicide Attempt (v2) Definition Factsheet & Technical Brief

^§^ Rounded to nearest whole number.

^¶^ Visit ratio = (ED visits for suspected suicide attempts [comparison group] / All ED visits [comparison group]) / (ED visits for suspected suicide attempts [Reference] / All ED visits [Reference]).

** Percent change was calculated using the following equation: ([Comparison visit proportion for 2022, 2023, 2024, or 2025 – Reference visit proportion for 2021] / [Reference visit proportion for 2021]) × 100. Changes were calculated with unrounded visit proportions.

†† Statistically significant difference between comparison and reference periods.

## Discussion

After reports of increases in U.S. ED visit numbers and visit proportions for suspected suicide attempts during 2020 and 2021 (*3*,*5*), this analysis of current data found an overall decrease in the proportion of suspected suicide attempt–related ED visits during 2021–2025. In addition to substantial decreases in visit proportions, decreases in visit numbers among adolescents aged 12–17 years also occurred during this period. Although these declines are encouraging, adolescents, particularly girls, continue to be disproportionately represented among ED visits for suspected suicide attempts. This report also noted that adults aged ≥26 years experienced small yet significant increases in proportions of ED visits for suspected suicide attempts from 2021 to 2025. Suicide attempts are more likely to be lethal with increasing age, especially among adults aged ≥65 years (*6*).

No single cause of suicide attempts exists (*7*). Risk factors for suicide attempts include, but are not limited to, job or financial problems, loss of relationships, and violence victimization; protective factors include access to health care, feeling connected to others, and having effective coping and problem-solving skills, among others (*7*). These and other factors might have varied during the study period and contributed to differential changes by group. For example, adolescents, especially girls, reported high levels of disconnection during periods of disruption to routines associated with the COVID-19 pandemic, which abated later in the study period (*8*). In contrast, older adults were disproportionately represented among those who had lost longer-term sources of social support, including close family members and friends, during the study period (*9*). However, these are just a few of many factors that might explain observed differences. In addition, youth suicide prevention measures, such as the 2021 Surgeon General’s Advisory on Protecting Youth Mental Health, have been an area of focused public health activity in recent years (*10*) and might have helped reduce suicide attempts.

### Limitations

The findings in this report are subject to at least six limitations. First, findings underestimate the number of suicide attempts, because not all suicide attempts, including those resulting in death and those among youths,^††^ are treated in ED settings; reductions in care-seeking behaviors might have further reduced the number of ED visits in the early parts of the study period, which coincided with the pandemic. Second, whether ED care-seeking behavior for suicide attempts was similar to that for other conditions during this period is unclear, which would affect observed visit proportions. Third, unavailability of population sizes for ED catchment areas precluded calculations of ED visit rates per population. Fourth, underascertainment of suicide attempts is likely given the administrative nature of the data and reliance on limited documentation in medical records. Fifth, findings of this study cannot be directly compared with those from studies using different definitions of suspected suicide attempts. Finally, this analysis did not examine state or local trends that could help guide prevention strategies to reduce suicide attempts and related behaviors.

### Implications for Public Health Practice

The number of ED visits for suspected suicide attempts remains high; therefore, prevention of suicide and suicide attempts in all persons, especially in groups with high or increasing proportions of ED visits for suspected suicide attempts, is an important consideration when planning public health activities. This prioritization includes incorporating less commonly examined groups, including adults aged ≥26 years who have experienced sustained increases in recent years, as well as adolescents, for whom suspected suicide attempts remain high despite decreases. Further, timely monitoring of trends, especially considering increases during 2024–2025 in youths and adolescents, is important to facilitating prompt action. Strategies implemented as part of a comprehensive approach, such as through CDC’s Comprehensive Suicide Prevention Program, are crucial to reducing suicide and suicide attempts. This approach is further detailed in CDC’s Suicide Prevention Resource for Action (*7*) and the 2024 National Strategy for Suicide Prevention. This comprehensive approach incorporates downstream prevention measures that can support persons in crisis^§§^ and includes strategies that can be implemented in ED settings such as the Zero Suicide framework. However, upstream strategies, such as promoting connectedness, teaching coping and problem-solving skills, and strengthening economic supports, can be critically important to addressing population-specific factors and preventing persons from becoming suicidal in the first place. Together, upstream and downstream prevention are essential for reducing suicide attempts and suicides.

## Footnotes

* These authors contributed equally to this report.

† Based on age-specific estimates (age groups 12–17 and >18 years); summed to produce total population estimate.

§ VR = (ED visits for suspected suicide attempts [comparison group] / All ED visits [comparison group]) / (ED visits for suspected suicide attempts [Reference] / All ED visits [Reference]). A 95% CI for a VR that excludes 1 indicates that the VR is statistically significant.

¶ Percent change was calculated using the following equation: ([Comparison visit proportion for 2022, 2023, 2024, or 2025 – Reference visit proportion for 2021] / [Reference visit proportion for 2021]) × 100. Changes were calculated with unrounded visit proportions.

** 45 C.F.R. part 46.102(l)(2), 21 C.F.R. part 56; 42 U.S.C. Sect. 241(d); 5 U.S.C. Sect. 552a; 44 U.S.C. Sect. 3501 et seq.

†† In 2023, 9% of high school students reported attempting suicide in the previous year; 2% reported being injured in a suicide attempt that resulted in an injury, poisoning, or overdose that had to be treated by a doctor or nurse during the previous year. Youth Risk Behavior Survey Data Summary & Trends Report: 2013-2023

§§ For persons in crisis, help is available through the Substance Abuse and Mental Health Services Administration’s 988 Suicide and Crisis Lifeline (https://www.988lifeline.org) or by texting or calling 988.
